# Fungal Prosthetic Joint Infection by *Candida parapsilosis* After Total Knee Arthroplasty

**DOI:** 10.3390/antibiotics14111112

**Published:** 2025-11-05

**Authors:** Zmago Krajnc, Klemen Žitek, Nina Gorišek Miksić

**Affiliations:** 1 University Department of Orthopaedics, University Medical Centre Maribor, 2000 Maribor, Slovenia; zmago.krajnc@guest.arnes.si; 2 Department of Orthopaedics, General Hospital Murska Sobota, 9600 Murska Sobota, Slovenia; klemen.zitek@sb-ms.si; 3 Department of Infectious Disease and Febrile Conditions, University Medical Centre Maribor, 2000 Maribor, Slovenia

**Keywords:** *Candida parapsilosis*, total knee arthroplasty, fungal prosthetic joint infection, antifungal spacer, case report

## Abstract

Total knee arthroplasty is an increasingly common surgical intervention for degenerative knee disease, yet it carries a risk of prosthetic joint infection (PJI). While bacterial infections dominate the landscape of PJIs, fungal infections represent a rare but significant concern, especially in immunocompromised patients. This case report describes a 71-year-old patient who presented in October 2024 with left knee pain and swelling 7 months after total knee arthroplasty. A prosthetic joint infection due to *Candida parapsilosis* was diagnosed preoperatively by repetitive microbiological examination of synovial fluid and intraoperatively by tissue samples and sonication of prosthetic material. A two-stage revision surgery with a short 4-week interval was performed using an antifungal-impregnated spacer, followed by 6 months of systemic antifungal treatment with fluconazole and continued by fluconazole suppressive treatment for another 6 months. A favorable clinical and functional outcome was achieved after 11 months of follow-up. This is a rare case of fungal PJI treatment with a two-stage revision with a shorter interval, using an antifungal impregnated spacer combined with a prolonged antifungal therapy.

## 1. Introduction

The number of total knee arthroplasty (TKA) surgeries for treating degenerative diseases of the knee joint has increased in recent years and continues to rise [1]. Prosthetic joint infection (PJI) is a challenging and devastating complication of TKA and one of the most common cases for revision surgery. The most common causative microorganisms are Gram-positive bacteria, predominantly *Staphylococcus aureus* and *Staphylococcus epidermidis*; although less prevalent, fungal infections, particularly those caused by *Candida* species, can also cause PJI [2]. Incidence of PJI after joint replacement surgery is 1–2%, of which fungal infections represent 1%. The most commonly isolated fungi are *Candida albicans*, followed by *Candida parapsilosis*, *Candida glabrata*, and *Candida tropicalis* [3]. Fungal PJIs are particularly common in immunocompromised patients, with risk factors including malignancy, diabetes mellitus, hormonal therapy, and many more. Previous surgeries on the affected joint are also considered a risk factor for infection [4].

*Candida parapsilosis* is considered a skin commensal organism that can become pathogenic under certain conditions. The major virulence factor of *Candida parapsilosis* is the ability to adhere and to form biofilm on biotic and abiotic (artificial) surfaces. Biofilm protects fungi against the immune system, contributes to resistance to antifungals, and facilitates persistence on surfaces (prosthetic joints), although the biofilm of *Candida parapsilosis* is less complex than that of *Candida albicans* on prosthetic surfaces [5,6]. Biofilm-related fungal infections are usually difficult to treat and necessitate a multifaceted approach that includes antifungal therapy and surgical intervention in the case of PJI [7,8]. Overall, *Candida parapsilosis* PJI had better outcomes (over 90% success rate) compared to *Candida albicans* PJI (with 50% success rate) according to the recent systematic review by Grzlecki et al. [9].

This case report represents a patient who developed a PJI due to *Candida parapsilosis* following TKA. The patient was successfully treated with two-stage revision surgery and both systemic and local antifungals. The case report presents a short interval treatment that is rarely described in fungal PJI, with a detailed description of the antifungal spacer composition that is also rarely described in detail in the literature, despite widely accepted use [9,10]. The case report adheres to reporting guidelines (CARE checklist in Supplemental Materials), and the patient gave written informed consent for case presentation.

## 2. Case Report

**Figure 1:**
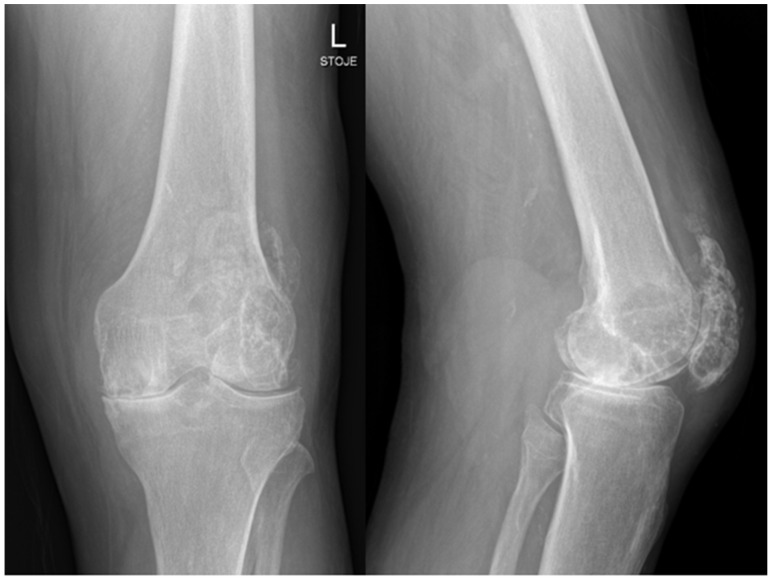
Radiology of left knee—anteroposterior and lateral views of the knee before implantation of total knee endoprosthesis, showing severe knee osteoarthrosis with heterotopic ossifications after quadriceps rupture and surgical treatment years ago.

A 71-year-old man with prostate cancer on hormonal therapy, chronic heart disease, hypertension, and hyperlipidemia underwent a primary left knee arthroplasty due to secondary post-traumatic osteoarthritis in February 2024. The patient had a knee arthroscopy in 2012 and reconstruction of the quadriceps tendon in 2014 on the same knee. The primary knee replacement was performed in February 2024 and was particularly challenging because of a large degenerative cyst in the lateral femoral condyle and a weakened quadriceps tendon with multiple foci of ossification. Preoperative severe osteoarthrosis of the left knee is presented on radiography in Figure 1.

**Figure 2:**
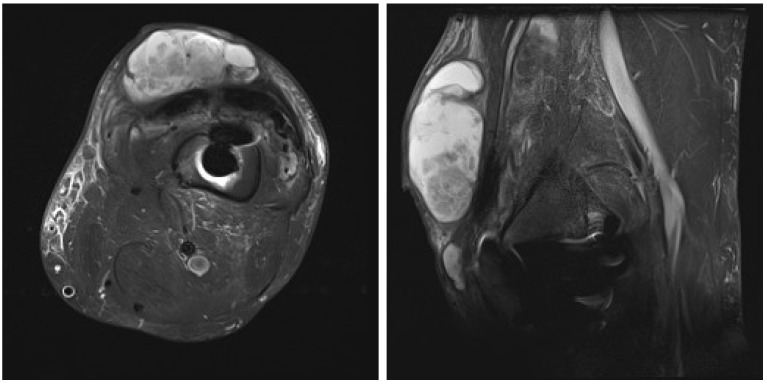
Contrast-enhanced magnetic resonance image of left knee: axial and lateral view of the left knee showing pronounced synovitis intra-articular with a small effusion, extending through the defect of the quadriceps tendon just above the patella into the subcutaneous tissue suprapatellar, forming a large septate collection, small osteolysis around the femoral part of knee endoprosthesis.

After an uneventful period of seven months, the patient was admitted in September 2024 with left knee pain, prepatellar swelling, and redness of the skin, without any systemic signs of infection. Laboratory tests revealed a normal complete blood cell count and an elevated C-reactive protein (CRP) level of 93 mg/dL. A high suspicion of PJI was established, and knee aspiration was performed twice in September 2024, with the samples sent to the microbiological laboratory. Laboratory analysis of the knee aspiration showed elevated leucocyte count in synovial fluid, with a differential cell count revealing 91% neutrophil granulocytes, total protein concentration of 44 g/L, consistent with an inflammatory process. Contrast-enhanced magnetic resonance imaging (MRI) of the left knee was performed, showing pronounced synovitis intra-articular with a small effusion extending through the defect of the quadriceps tendon just above the patella into the subcutaneous tissue suprapatellar into a large septate collection (Figure 2).

**Figure 3:**
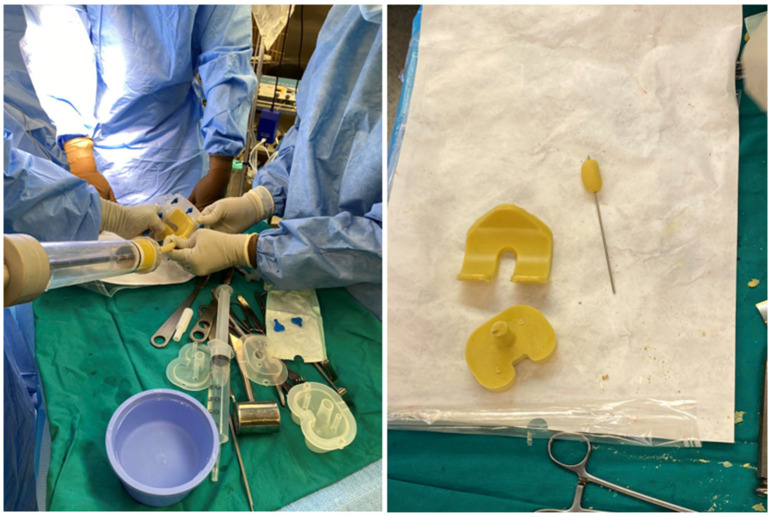
Preparation of antifungal-impregnated spacer.

**Figure 4:**
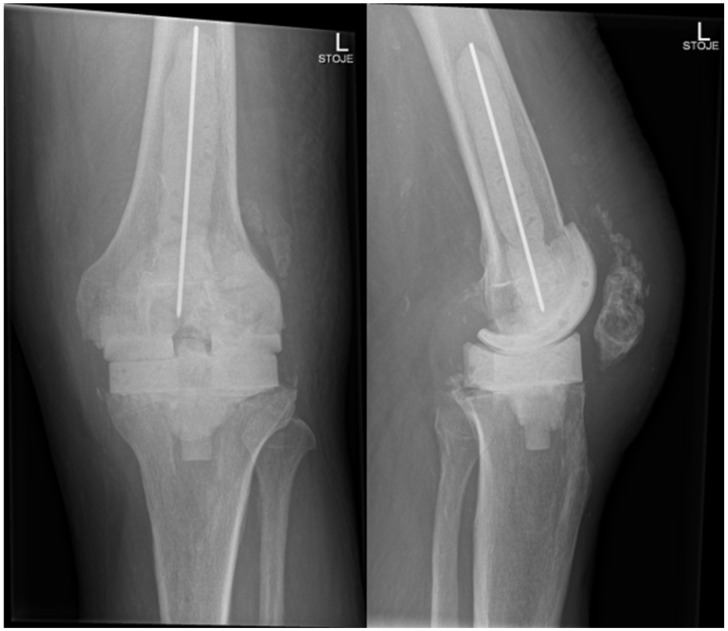
Radiography of the left knee—anteroposterior and lateral views of the knee after removal of knee endoprosthesis and insertion of an antibiotic- and antifungal-impregnated cemented spacer.

*Candida parapsilosis* grew in both synovial fluid samples from separate aspirations and was identified by standard microbiology methods at the microbiology laboratory at the National laboratory of health, environment and food, Maribor. Unfortunately, a mycogram was not obtained, as it is not performed in all samples routinely at the laboratory. According to the retrospective analysis of bloodstream fungal infection from central Slovenia, the *Candida parapsilosis* resistance rate to fluconazole was very low, and we decided to start the treatment preoperatively with oral fluconazole 400 mg/day [11]. A decision to perform a two-stage revision surgery with a 4-week interval was taken. The first stage was performed in October 2024, involving the complete removal of the implant, debridement, and insertion of an antibiotic- and antifungal-impregnated cemented spacer. For the spacer, we used Palacos R+G^®^ cement (Heraeus Medical GmbH, Wehrheim, Germany), with each package containing 40.8 g of cement powder, 20 mL of monomer liquid, and 0.5 g of industrially premixed gentamicin. Additionally, we supplemented it with 0.25 g of non-liposomal amphotericin B deoxycholate powder (Fungizone^®^, Delpharm, Boulogne-Billancourt, France) per package. A total of three packages were used, resulting in a cumulative amphotericin B dose of 0.75 g. The cement powder, antifungal powder, and monomer liquid were thoroughly blended by hand, resulting in a yellowish-colored bone cement of proper hardness. In Figure 3, the preparation of an antifungal-impregnated cemented spacer is shown. During the operation procedure, 5 intraoperative tissue samples, synovial tissue samples, 2 bone tissue samples with residual non-resorbable suture material, and the explanted prosthesis were collected for further microbiological analysis; sonication of the explanted material was performed and sonication fluid cultured according to a standardized protocol. *Candida parapsilosis* grew from all tissue samples, bone samples, and from the sonication fluid of the removed implant in low concentration, 3 CFU/mL. Tissue samples were also sent for histopathology exam, and acute inflammation was confirmed. Antifungal therapy with intravenous caspofungin (70 mg) was started preoperatively, then 50 mg/day for two weeks, followed by an oral switch to fluconazole at 400 mg/day. The patient continued fluconazole therapy until the second stage of surgery, with no drug holiday before reimplantation was planned. In Figure 4, the left knee is presented in radiography after the first-stage surgery with insertion of an antibiotic- and antifungal-impregnated cemented spacer.

**Figure 5:**
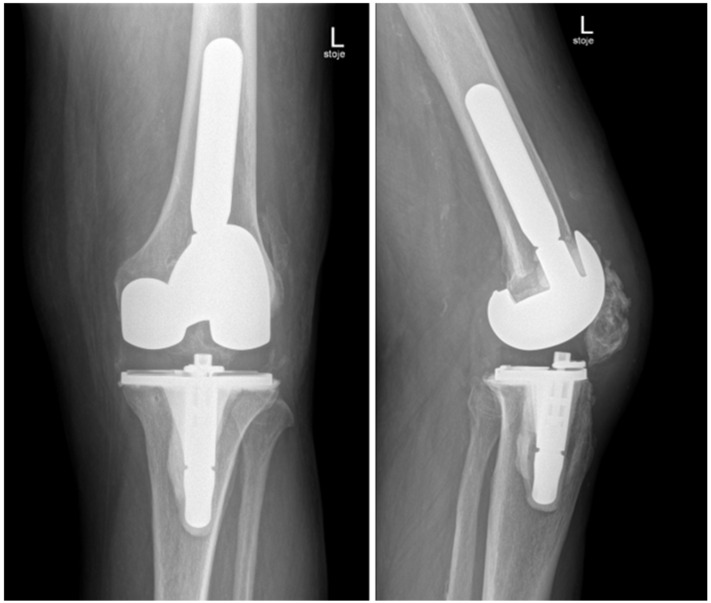
Radiography of the left knee—anteroposterior and lateral views after reimplantation of revision knee endoprosthesis.

In November 2024, four and a half weeks later, the second stage was performed, involving the removal of the spacer and reimplantation of the revision prosthesis. The reimplantation procedure was a complex and surgically demanding procedure. A significant bone defect, in combination with a degenerative cyst in the lateral femoral condyle, required careful management during the reimplantation procedure. The cyst added complexity to the structural integrity of the femoral condyle, demanding attention to ensure proper fixation of the new prosthesis. Additionally, wound closure proved difficult, since it was impacted by scar tissue resulting from the previous two surgeries. Moreover, the patient had an attenuated quadriceps tendon, which added to the challenges of achieving stable joint function post-revision. The weakened tendon posed a risk for compromised rehabilitation and further functional limitations. Tissue samples, including synovial tissue and a portion of patellar bone with residual non-resorbable suture material and spacer, were once again obtained for microbiological analysis. The spacer was sonicated, and the sonication fluid was cultured. Preoperatively, the patient received 70 mg intravenous caspofungin, piperacillin/tazobactam at 4.5 g every 8 h, and 1–1.5 g of vancomycin every 12 h (depending on serum levels) until the microbiological results of the intraoperative samples were available. Tissue samples remained sterile, and after two weeks, the patient was discharged home on oral fluconazole therapy (400 mg/day) for the next 6 months. A prolonged suppressive antifungal therapy is planned thereafter (200 mg daily for the next 6–12 months). Upon discharge, inflammatory parameters were in decline (CRP 13 mg/dL), and the surgical wound was adequately healed. At the last follow-up check-up 11 months after the surgery in October 2025, the patient’s knee was painless, without effusion, and the surgical wound healed. The knee mobility was from 0 to 100 degrees, and greater flexion is not expected due to the quadriceps scar. The patient is pain-free and can handle all daily loads. The Knee injury and Osteoarthritis Outcome Score (KOOS) is 84. Inflammatory markers remained negative, indicating no signs of infection or ongoing inflammation. The patient experienced no side effects from antifungal therapy. He is still on fluconazole 200 mg orally, but the suppressive therapy will be stopped in 1 month (12 months after surgery). Further careful clinical monitoring is planned. This clinical progression suggests a favorable post-revision outcome, with the patient tolerating the suppressive antifungal treatment well. The patient is satisfied with the treatment result. In Figure 5, radiography after the reimplantation of the revision endoprosthesis is presented.

## 3. Discussion

This case report describes a patient with multiple comorbidities who developed a delayed onset PJI due to *Candida parapsilosis* following TKA. Fungal PJI is rare, and the presence of immunosuppressive conditions, such as hormonal therapy, malignancy, and diabetes mellitus, likely contributes to the susceptibility to infection [12]. Our patient has a medical history of prostate carcinoma treated with hormonal therapy and a history of two previous surgeries on the affected knee joint. These two risk factors likely played a significant role in the development of the infection [13].

Clinical signs of fungal PJI are usually of a low-grade infection, such as in this case, where the patient presented with knee pain, prepatellar swelling, and redness of the skin. Joint aspiration is a diagnostic cornerstone in low-grade PJI and yields positive cultures; nevertheless, the literature suggests that cultures can often be negative. However, an elevated leucocyte count and standard diagnostic procedures for PJI help in the diagnosis of PJI [14]. Due to the rarity of fungal PJI, it is crucial to have a proper microbiological diagnostic in both the preoperative and intraoperative phases, with tissue samples and sonication of removed implants and sonication fluid culture. The importance of preoperative joint aspiration in PJI diagnostics in our case is shown by the fact that we were able to adapt our therapeutic and surgical plan according to the isolate. A major limitation of our case is the lack of mycogram in *Candida parapsilosis*, although we are well aware of the rising fluconazole resistance in *Candida parapsilosis* [15].

The identification of *Candida parapsilosis* prompted the decision for a two-stage revision, which is widely suggested in the literature as the preferred treatment approach for fungal PJIs [16]. The first stage included removal of the infected implant and placement of an antibiotic- and antifungal-impregnated spacer. Evidence suggests that improved outcomes can be achieved when local antifungal agents are used directly at the site of infection [17]. Both non-liposomal amphotericin B (with deoxycholate) and liposomal amphotericin B can be mixed with cement for local delivery in the treatment of fungal PJIs since they are both stable at a temperature over 70 °C, which enables their usage in PMMA cement spacers. Studies suggest that liposomal amphotericin B has the strongest biofilm-prevention capacity and provides enhanced drug release from bone cement when compared to non-liposomal amphotericin, making it the preferred choice for treating fungal infections in prosthetic joints, although all forms of amphotericin B formulations were active against *Candida* biofilms [18,19]. We used a lower dose of non-liposomal amphotericin (0.25 g of amphotericin per 40 g Palacos R+G^®^ cement powder), since the recent analysis showed successful outcomes with lower doses as well [10]. However, we decided to use cement with gentamicin added since substances like gentamicin act like poragens, which improve elution of amphotericin from cement at the infection site [10,14]. A study by Kweon et al. suggested that using gentamicin as a poragen improves the release of non-liposomal amphotericin B. However, it decreases the compressive strength of the cement, limiting its use for implant fixation [20]. The study by Czuban et al. found that the addition of gentamicin did not improve the release of deoxycholate amphotericin B and also did not lower compressive strength [19]. In our case, there was no significant concern regarding compressive strength, as the cement was intended solely as a spacer and not for fixation. The ratio of amphotericin B to gentamicin (poragen) used in our case was 1:2, differing from the ratios in previous studies, where Kweon used a 1:50 ratio and Czuban used up to 1:4 [19,20]. We used commercially prepared PMMA cement with a fixed dose of gentamicin.

A regimen of intravenous caspofungin pre- and post-operatively, followed by oral fluconazole, corresponds to current antifungal protocols for treatment of fungal PJI [21].

There is no consensus about the optimal time of reimplantation in fungal PJI. A study by Phelan et al. involving 164 patients reported a median interval of 8.6 months for total hip arthroplasties and 2.3 months for total knee arthroplasties between implant removal and reimplantation procedures [22]. Another retrospective study involving fungal PJI found a median duration of 33.9 weeks between implant removal and reimplantation. This study also highlighted that a longer interval and the presence of mixed bacterial infections were associated with higher treatment failure rates [23]. The most recent publication by Tan et al. described an interval of 4 weeks or less (3 weeks) in 2 cases among 102 [10]. In our case, performing reimplantation after four and a half weeks resulted in favorable outcomes, including a decline in inflammatory markers and proper wound healing. Additionally, all tissue samples and sonication of the spacer collected at reimplantation remain sterile, suggesting effective local and systemic treatment. This suggests that a shorter interval may be effective, provided that proper infection control measures are implemented. However, it is crucial to tailor the timing of reimplantation to each patient’s unique circumstances, considering factors such as the organism involved, patient health status, and response to initial treatments. Patients’ inconvenience, costs, and length of hospital stay are significantly reduced in a shorter interval, and it is essential to consider this when planning the therapeutic protocol [24].

Regarding the systemic antifungal therapy in PJI, it is important to choose an antifungal with good biofilm activity, where amphotericin and echinocandins both have good activity against biofilm [25]. Unfortunately, they are not available in an oral formulation that would enable a shorter hospital stay. We treated the patient with biofilm-active antifungal caspofungin for the first two weeks after implant removal, when infected tissue was debrided surgically, but since a spacer was used in the possibly contaminated field, we decided to treat the patient for 2 weeks systemically with caspofungin, which should prevent biofilm formation on the spacer, combined with local amphotericin delivery from the spacer. After 2 weeks, we continued with oral fluconazole until reimplantation, when again, the patient received caspofungin for 14 days and was later discharged home on fluconazole. The type and duration of suppressive antifungal therapy after the second stage of surgery are also unclear. A long-term study of Candida species demonstrated that fluconazole has appropriate antifungal efficacy for suppressive treatment in implant-related fungal infections [26]. Sustained antifungal therapy for 6 months reflects a common approach, highlighting the importance of prolonged treatment to prevent recurrence of infection [27]. According to the Pocket Guide to Diagnosis & Treatment of PJI, PRO-IMPLANT Foundation, up to 12 months of suppressive antifungal therapy is recommended in the case of fungal PJI. Since the patient tolerated fluconazole therapy well, we decided to prolong the therapy to 12 months after surgery [28].

## 4. Conclusions

This case illustrates a case of fungal PJI infection and highlights the importance of preoperative microbiological diagnostics and effective therapeutic planning, incorporating comprehensive surgical and pharmacological strategies. A multidisciplinary, patient-adapted approach is crucial in treating PJI that is difficult to manage to achieve the desired outcome. Two-stage revision surgery in combination with prolonged antifungal therapy seems to be the therapeutic option of choice, and a shorter interval can be successful.

## Data Availability

The data presented in this study is available on request from the corresponding author due to patient personal data protection.

## Supplementary Materials

The following supporting information can be downloaded at: https://www.mdpi.com/article/10.3390/antibiotics14111112/s1. File S1: CARE checklist.
